# Risk Factors for Hearing Loss in Children following Bacterial Meningitis in a Tertiary Referral Hospital

**DOI:** 10.1155/2013/354725

**Published:** 2013-05-15

**Authors:** Benson Wahome Karanja, Herbert Ouma Oburra, Peter Masinde, Dalton Wamalwa

**Affiliations:** ^1^University of Nairobi, P.O. Box 2209-00202, KNH, Nairobi, Kenya; ^2^Department of Surgery, University of Nairobi, P.O. Box 30197-00100, G.P.O. Nairobi, Kenya; ^3^ENT Department, Kenyatta National Hospital (KNH), University of Nairobi, P.O. Box 20723-00202, Nairobi, Kenya; ^4^Department of Pediatrics and Child Health, University of Nairobi, P.O. Box 19676-00202, Nairobi, Kenya

## Abstract

*Objective*. This study aimed to examine hearing function in children admitted with bacterial meningitis to determine the risk factors for sensorineural hearing loss. *Setting*. The study was conducted in the audiology unit and paediatric wards of Kenyatta National Hospital. *Subjects and Methods*. The study involved 83 children between the ages of six months and twelve years admitted with bacterial meningitis. The median age for the children examined was 14. On discharge they underwent hearing testing to evaluate for presence and degree of hearing loss. *Results*. Thirty six of the 83 children (44.4%) were found to have at least a unilateral mild sensorineural hearing loss during initial audiologic testing. Of the children with hearing loss, 22 (26.5%) had mild or moderate sensorineural hearing loss and 14 (16.9%) had severe or profound sensorineural hearing loss. Significant determinants identified for hearing loss included coma score below eight, seizures, cranial nerve neuropathy, positive CSF culture, and fever above 38.7 degrees Celsius. *Conclusions*. Sensorineural hearing loss was found to be highly prevalent in children treated for bacterial meningitis. There is need to educate healthcare providers on aggressive management of coma, fever, and seizures due to their poor prognostic value on hearing.

## 1. Introduction

Deafness is one of the commonest serious complications of bacterial meningitis in childhood. In developed countries, approximately 10% of survivors of bacterial meningitis are left with permanent sensorineural hearing loss [1–3]. Other children experience a transient hearing loss [3–6]. Both types of hearing impairment are thought to develop during the first few days of the illness [5–7].

Kenyatta National Hospital, KNH, is Kenya's national referral hospital. Estimates show that an average forty-five children are admitted into its pediatric wards each month with a confirmed diagnosis of bacterial meningitis.

Behavioral tests of hearing may be used when an infant reaches the developmental age (as opposed to the chronological age) of six months. Infants not at this level of development and some of those with more than one disability will need to be tested by otoacoustic emissions (OAEs) and auditory brainstem responses (ABRs). Unfortunately the equipment for these latter two sets was unavailable forcing the study to be carried out in children above 6 months of age using behavioural distraction testing.

So far, in KNH no similar study had been undertaken to determine the prevalence, burden, and risk factors for hearing loss following bacterial meningitis in children admitted to KNH. 

## 2. Materials and Methods

### 2.1. Participants

The study involved 83 children (49 males and 34 females) between the ages of six months and twelve years admitted with bacterial meningitis from the pediatric wards, KNH. All cases admitted within twenty-four hours of diagnosis, who met the inclusion criteria, were recruited every weekday evening during the 3-month study period.

All participants fulfilled the following criteria: age of six months or older at the time of admission and confirmed diagnosis of bacterial meningitis. Bacterial meningitis was defined according to the World Health Organization (WHO) workbook recommendations based on laboratory findings, symptoms, or signs [8]. Those excluded from the study included all subjects with a confirmed diagnosis of tuberculosis and those on current treatment for tuberculosis; those with a prior history of hearing loss; those using ototoxic antibiotics as part of treatment; those with chronic medical conditions (diabetes, renal, cardiac diseases); those on treatment for malaria.

A full medical history was documented and factors relating to the patient and prior treatment documented by the principal investigator in the questionnaire. The history included the parents or guardians' assessment of hearing and any history of ear discharge or infection. However, there were no premeningitis audiograms, which is a potential confounding factor and weakness of the study. Otoscopy was done using a Riester hand-held otoscope and its aural speculums and the findings recorded. The children then underwent a thorough physical examination. Hematological and CSF study results were documented. The Glasgow Coma Scale, GCS, was used for the level of consciousness. All these findings were entered in the patient's data entry form. 

The relevant hearing assessment was done prior to discharge from hospital and two weeks after. This identified and excluded transient hearing loss. 

All participants' parents or guardians gave informed consent for the study. Five guardians declined to have their children involved in the study. The study protocol was approved by the institutional ethics and review committee of KNH.

### 2.2. Audiological Protocol

All patients completed age-appropriate hearing tests carried out by trained audiologists in the ENT audiology section in a sound- proofed booth. This was well lit with minimal littering to minimize the child's distraction. It was also well ventilated and large enough to accommodate the child and parent/guardian, tester, and distractor. The standard ambient noise was 35 dB.

Children between six and twenty-four months of age underwent the distraction test, those between twenty-four and thirty-six months of age had the performance tests done, while pure tone audiometry was carried out on children above thirty-six months of age. They were carried out in the following manner.

(1) *Behavioral Testing*. The behavioral testing equipment included toys that were not too bright used by the distractor to distract the child being examined. Three warblers were used: low, mid, and high frequency types. A Manchester rattle was also used to deliver high frequency sounds during behavioral testing. The G-chime was used for mid-frequency sounds and the C-chime for high frequency sound generation.*Distraction Test*. A distraction test was performed if the infant was sitting and able to turn and locate the source of a sound. It was carried out with the infant sat upon an adult's knee facing forwards where a distractor controlled the infant's attention using toys. The tester introduced the sound signals at high, mid, and moderate frequencies from 45 degrees and one meter behind, at the level of the ear. These were tested separately in order to detect hearing loss restricted to one part of the frequency range. The sounds were introduced at very quiet levels (35 dBA). Care was taken not to give clues as to the tester's position other than the test signal.*Performance Tests.* The child was shown how to wait until a sound was heard before carrying out an action. Once this could be done, the test was to be performed at a meter distance and from behind. The test was performed using “Go” for low frequencies, “S” for high frequencies, introduced at the quietest voice levels or 2 Warble tones at 500 Hz, 1, or 2 kHz, and 4 kHz introduced at a very quiet level corresponding to normal hearing. The child was said to have “passed” the screen if there were two responses at the quietest level. 

(2) *Pure Tone Audiometry*. This was carried out in all children recruited for the study who were above thirty-six months of age. Pure tones (20 dD Hearing Level, HL) were introduced using headphones and testing carried out by air conduction (500 H–4000 Hz) and bone conduction (500–4000 Hz). Pure tone audiometry was performed using an Interacoustics clinical audiometer (Model AC33; serial no. SN735530, calibration date June 2009). Sound delivery and masking during pure tone audiometry was ensured using TDH-399 headphones.

The main outcome measure was presence or absence of sensorineural hearing loss in the children.

### 2.3. Followup

If the child had made a complete recovery from meningitis, lived far from the hospital, and had no hearing loss, followup was not done.

If the child had sequalae that required further management beyond 2 weeks, followup was continued. For those children exhibiting hearing loss, whether conductive or sensorineural, recommendations for management and followup were based on specific test findings and varied accordingly.

## 3. Statistical Analysis

Descriptive statistics (means for continuous variables and proportions for categorical variables) were calculated to describe the population. 

The main outcome was degree of sensorineural hearing loss as measured by the age-appropriate hearing test. This had three levels of outcomes: normal, mild/moderate, and severe/profound. The population proportions and the 95% confidence interval, CI, were estimated for each category of outcome, using statistical methods to give more precise estimates. All categorical variables were cross-tabulated with the outcome and Pearson Chi Square computed. 

## 4. Results

**Figure 1:**
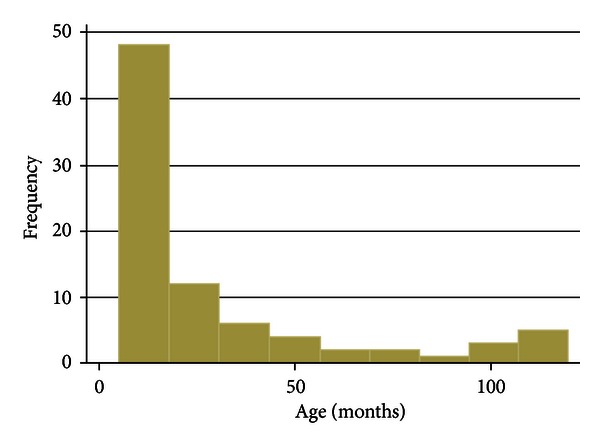
Age distribution (in months) of children evaluated.

**Figure 2:**
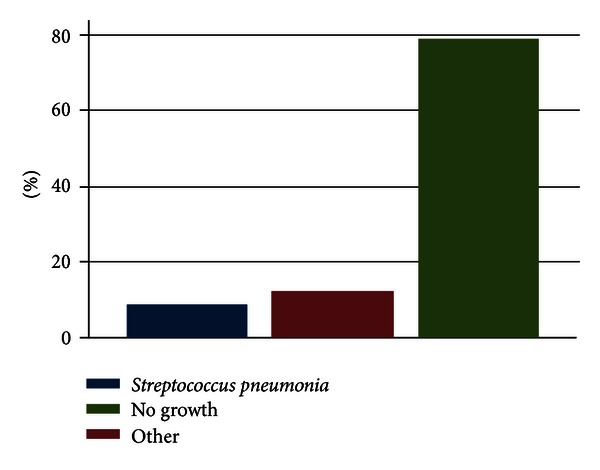
Bar chart of cerebrospinal fluid culture findings.

The outcomes of eighty-three children (49 males and 34 females) admitted with bacterial meningitis during the study period were analyzed.  Figure 1 shows the age distribution (in months) of children evaluated. The median age for the children examined was 14 months (range from 5 to 120 months) as shown on Table 1. The characteristics of the children studied are presented in Table 2. A minority of the children were malnourished (15/83; 18.1%) and almost all caregivers (82/83; 99%) had some form of education. More than two thirds of the children presented with fever (54/83; 65%) and rarely had a cranial nerve palsy (8/83; 10%) or hydrocephalus (5/83; 6%). See Table 2 and Figure 2. Only two in ten cerebrospinal fluid culture samples yielded a growth with most being *Streptococcus pneumoniae *(10/17) (Figure 2). 

On CSF microbiology, three categories were included:

1. no cultured organism;
2. *Streptococcus pneumoniae*  (10);
3. other  (7)
  - *Neisseria meningitidis*—4,
  - *Haemophilus influenzae*—3.

Only seventeen (20.5%) of CSF specimens examined cultured any bacteria. *Streptococcus pneumoniae, Haemophilus influenza*, and *Neisseria meningitidis* were isolated in 10, 4, and 3 children, respectively. The latter two were classified as “other” microorganisms in comparison with *S. pneumoniae. *There were no distinctly different clinical presentations among children with *S. pneumoniae, H. influenza*, and *N. meningitidis* meningitis. CSF culture findings are illustrated in Figure 2. 

A significant number of patients enrolled developed sensorineural hearing loss as a sequalae (36/83). The overall prevalence of SNHL loss was estimated using bootstrap with 1000 repetitions to be 43.37% (95% CI: 33.22, 55.93). Of the children with hearing loss, 22 (26.5%) had mild or moderate sensorineural hearing loss, and 14 (16.8%) had severe or profound sensorineural hearing loss. The prevalence of specific categories of SNHL is presented in Table 3.

The results of the univariable ordinary logistic models are presented in Table 4. Comparing those who had normal hearing to those who have any SNHL, there is strong evidence to suggest that having a seizure or a positive CSF microbiological culture increased the odds of having SNHL (Model 1 on Table 5). Similarly, history of seizure or a positive culture was strongly associated with having moderate SNHL (Model 1 on Table 5). None of the predictors was significantly associated with severe SNHL. This finding may be as a result of the small numbers involved in this analysis and therefore low power to detect differences. 

Table 6 shows the results of final ordinary logistic models fitted with variables significant from the univariable analysis. Overall after controlling the effect of sex and age, a positive CSF microbiological culture and history of seizure strongly predicted SNHL.

In the univariable multinomial logistic regression agitation, fever, seizure, and a positive CSF culture were associated with moderate SNHL, while only seizures were associated with severe SNHL. Estimates could not be obtained for some variables as there were too few numbers within the various categories of the variable.

From the final multivariable multinomial regression, seizures and positive CSF culture predicted both moderate and severe SNHLs, while agitation predicted moderate SNHL.

 Interestingly, the ordered logistic models allowed for the inclusion of more variables which were otherwise dropped out of other models. After adjusting for age and sex, these models suggest that seizures, positive CSF culture, agitation, presence of cranial nerve palsy, and a coma score below eight strongly predicted both moderate and severe SNHL. These models probably provide the most efficient and consistent results. The assumption of proportional odds was not violated by any of the ordered logistic models fitted. Thus, an assumption that there was some ordering in the severity of SNHL seems sensible and appropriate. That is to say that moderate SNHL is proportionally worse than normal, and severe SNHL is proportionally worse than moderate SNHL. Thus, the models provide, for instance, the ordered odds ratio estimate of comparing those with positive CSF culture with those with no growth given that they had a certain severity of SNHL, when other variables are held constant. In this example, those with a positive culture are at an eightfold increased odds of having moderate SNHL compared to those with a negative culture, after controlling for other factors.

Age, nutritional status, gender, number of siblings, caregiver level of education, length of illness prior to admission, and stiff neck were not found to be significant risk factors for hearing loss.

All children with positive culture for *S. pneumoniae* and *H. influenzae* developed hearing loss, while two of three children with positive culture for *N. meningitidis* developed hearing loss.

Lowered CSF glucose and elevated CSF protein were both found to influence development of hearing loss although this did not reach statistical significance.

## 5. Discussion

In the present study, children proven by history and CSF findings to have bacterial meningitis were evaluated and found to have a sensorineural hearing loss prevalence of 43.4% (95% CI). This was greater than findings of previous studies. Kutz et al. reported an incidence of 14% [8] consistent with other reports [2, 4, 9, 10]. This is likely due to several factors. The study institution is a tertiary referral center for a major metropolitan area that understandably receives a disproportionate number of very sick children. In addition, perhaps current pathogens are more virulent owing to continued drug resistance. Finally, most children in this study had not had previous objective audiologic testing, and a negative history for hearing loss was based on history alone. Therefore, a few of them may have had a previously undiagnosed hearing loss despite attempts at identifying that by the principal investigator using the questionnaire. This could potentially inflate the prevalence of hearing loss; however, it is unlikely that this would significantly affect the overall prevalence. 

Consistent with most prior studies, this work did not reveal any relationship between occurrence and severity of hearing loss to the male gender. Forty-nine (59%) of the children evaluated were males, while 34 (41%) were females. There were no differences in the prevalence of hearing loss between the two groups. Kutz et al. showed that being male was a significant independent risk factor for hearing loss [8].

Early age at illness was identified by Grimwood et al. as a significant risk factor for hearing loss, with children suffering from meningitis before twelve months of age performing more poorly than children suffering from meningitis later in infancy and childhood, as well as age-matched controls, on measures of language and reading skills [10]. However, neither age nor sex was found to affect hearing outcome. This is in agreement with most previously reported studies [8, 9, 11–13].

Only seventeen (20.5%) of cerebrospinal fluid specimens were reported as positive for microorganism culture. The isolates, *Streptococcus pneumonia, Haemophilus influenza, *and* Neisseria meningitides*, were 59%, 23%, and 17.9%, respectively. A study in KNH revealed that in 82% of the cases, the cerebrospinal fluid cultures were bacteriologically positive [14]. Common isolates included *S. pneumoniae *(45%)*, N. meningitidis *(14%), and *H. influenzae *(12%). Other studies describe distinctly different clinical presentations between these different causative organisms. This studies' low pick-up rate may have reduced the power to determine the same. Fortnum found no differential risk of hearing impairment by causative agent in one study from Nottingham, UK [2].

All children with *S. pneumoniae* meningitis developed hearing loss. However, a very low proportion of CSF specimens (20.5%) cultured bacteria and so this may be an overestimate. Richardson et al. showed that the incidence of sensorineural hearing loss in children with *S. pneumoniae* meningitis was 36% [15]. Patients who developed hearing loss required longer hospitalization compared with patients who retained normal hearing.

Seizures and a coma score of less than eight were found to be the most significant determinants for hearing loss. Thirteen children (15.7%) had coma scores of less than eight on admission and all but one developed hearing loss. Seizures occurred in 64 (78%) of the patients. Woolley et al. found no correlation between hearing loss and seizures or hearing loss and altered level of consciousness [16]. Seizures are a common complication of meningitis in most studies occurring in 20%–30% of patients, but the cause of hearing loss in meningitis may be a different pathogenic process than the one that results in neurologic deficits [17]. In the large retrospective study by Woolley et al., 26% had seizures. Of children with hearing loss, 32% had seizures compared with 24% of those without hearing loss. Walter et al. showed that in patients who were found to have hearing loss, 45% also developed seizures. This rate is comparable with a seizure incidence of 25% in patients without hearing loss. Of the children with seizures and hearing loss, 69% developed at least severe hearing loss. Chang et al. demonstrated an overall worse prognosis for patients who develop seizures [18]. The development of seizures is multifactorial and may be due to high fevers, metabolic disturbances, or focal cerebral irritation. 

Concurrent cranial nerve neuropathy was found to be a strong predictor for the subsequent development of hearing loss, with all eight of the children (8/83; 10%) with cranial nerve neuropathy developing hearing loss. The only cranial nerve neuropathy found was Abducens nerve palsy. The presence of a cranial nerve neuropathy is certainly a sign of a severe infection and is highly correlated with the development of hearing loss. Of the patients with hearing loss and cranial nerve neuropathy, seventy-one had at least a severe sensorineural hearing loss. Kutz et al. found that 6% of the patients developed a cranial nerve neuropathy, and all but three were found to have hearing loss [8]. 

Decreased CSF glucose and elevated CSF protein as risk factors for hearing loss did not reach statistical significance. Decreased CSF glucose was the most consistent predictor of hearing loss, as illustrated by Woolley et al. and in previous studies [8, 16, 19]. It is unclear why a low CSF glucose level is such a strong predictor of hearing loss. It may be assumed that a low CSF glucose level correlates with high bacterial concentration of the CSF, increasing the likelihood of suppurative labyrinthitis as an etiology of subsequent hearing loss. However, other studies have demonstrated a much weaker association with elevated CSF protein and CSF pleocytosis, two factors that are elevated in patients with a high CSF bacterial concentration. In the study by Woolley et al., patients with hearing loss infected with *S. pneumococcus* were found to have a significantly higher CSF protein level. Another potential sequalae of low CSF glucose is direct damage to the cochlea neuroepithelium.

Presence on fever above 38.7 degrees was a significant risk factor for hearing loss with 22/54 children recording these high fevers developing hearing loss. Duration of illness beyond one week prior to admission and caregiver level of education were not found to be significant determinants for hearing loss following bacterial meningitis. Reports of the effect of delay in treatment on hearing outcome differ [11, 12, 18, 20–22]. If children with histories of more than seven days are excluded, as the symptoms were nonspecific and may be unrelated to the onset of meningitis, they found a nonsignificant increase in incidence of hearing loss in those with a longer history of fever (50% versus 36%, *P* = 0.13). Radetsky and Kilpi et al. found that the length of history of nonspecific signs and symptoms did not correlate with outcome [23, 24]. Kutz et al. found that length of hospitalization was a significant predictor of hearing loss [8]. 

Malnutrition was not found to have any significance in the development of hearing loss. Poor nutrition is associated with a poor outcome from meningitis, and a low weight for age was associated with a poor outcome in a study by Molyneux et al. [11].

## 6. Conclusions

Hearing loss is highly prevalent in children treated for bacterial meningitis in Kenyatta National Hospital with a prevalence of 43.4%. 

Strong risk factors for hearing loss following bacterial meningitis include coma score on admission of less than eight, development of seizures, concurrent cranial nerve neuropathy, positive CSF culture, and fever above 38.7 degrees Celsius. This is similar to the findings of studies done elsewhere.

Length of illness prior to admission was found not to be a determinant of hearing loss following bacterial meningitis. This is contrary to other studies done.

Age at illness, male gender, malnutrition, and primary caregivers' level of education were found not to be significant determinants of hearing loss following bacterial meningitis. The prior three were found in other studies to be significant predictors for the development of hearing loss.

Lowered CSF glucose and elevated CSF protein are minimally correlated with development of hearing loss following bacterial meningitis. This is different from other studies that place lowered CSF glucose high on the list of most significant predictors for hearing loss following bacterial meningitis.

There exists a need for objective hearing assessment in infants and young children following bacterial meningitis. This should be mandatory in all patients treated for bacterial meningitis.

## Figures and Tables

**Table 1:** Mean and median age ranges.

Mean (SD)	Median (range)
29.09 (31.71)	14 (5–120)

**Table 2:** Characteristics and findings in children with meningitis included in this study (*n* = 83).

Variable	Overall	Sensorineural hearing loss (SNHL)			
	*n*/*N* (%)	Normal (*n* = 47)	Mild/moderate (*n* = 22)	Severe/profound (*n* = 14)	Test
Biometrics					
Age in months (median, range)	14 (5–120)	15 (5–120)	10 (6–108)	14 (6–120)	*P* = 0.38*
Males	49/83 (59%)	29/47	13/22	7/14	*P* = 0.73

Anthropometric					
Weight, mean *z* score (SD)^*ξ*^	−0.89 (1.96)	−0.81 (2.10)	−1.24 (1.76)	−0.60 (1.84)	*P* = 0.59*
Malnourished (Yes)	15/83 (18.1%)	9/47	4/22	2/14	*P* = 0.91

*Clinical variables *					
History					
Seizures	64/82 (78.1%)	30/46	21/22	13/14	**P** = 0.006
Examination					
Fever (Y)	54/83 (65.1%)	32/47	17/22	5/14	**P** = 0.03
Level of consciousness (GCS)					
Coma score < 8	13/83 (15.7%)	1/47	9/22	3/14	
Coma score > 8	70/83 (84.3%)	46/47	13/22	11/14	**P** < 0.001
Cranial nerve palsy (Y)^†^	8/83 (9.6)	0/47	4/22	4/14	**P** = 0.002

*Laboratory *					
CSF culture					
No growth	66/83 (79.5%)	46/47	13/22	7/14	
Other	7/83 (8.4%)	1/47	4/22	2/14	
*Streptococcus pneumonia *	10/83 (12.1%)	0/47	5/22	7/14	**P** < 0.000
CSF biochemistry					
CSF glucose					
Median, range (g/dL)	29.7 (21.5–90)	31.2 (21.5–90)	27.7 (22.2–90)	27.2 (21.5–90)	*P* = 0.63*
>40 mg/dL	7/83 (8.4%)	2/47	4/22	1/14	*P* = 0.150
<40 mg/dL	76/83 (91.6%)				
CSF protein					
Median, range	225 (11–952)	231 (123–952)	222.5 (11–952)	189.5 (128–653)	*P* = 0.37*
<100 mg/dL	0/83 (0)	0	0	0	
>100 mg/dL,	83/83 (100%)	47/47	22/22	14/14	—

^†^All palsies involved the Abducens (Cranial nerve VI); ^*ξ*^based on CDC growth charts, US version 2000; *Levene's robust test statistic for the equality of variances between the groups.

GCS: Glasgow Coma Scale.

**Table 3:** Prevalence of SNHL.

SHNL category	Prevalence (%)	Normal based 95% CI
Normal	47/83 (56.62)	[44.79,66.05]
Mild/moderate	22/83 (26.51)	[17.33,35.68]
Severe/profound	14/83 (16.87)	[10.22,25.93]
Overall SNHL	36/83 (43.37)	[33.22,55.93]

**Table 4:** Univariable ordinary logistic regression models.

Variable		Model 1			Model 2			Model 3	
	OR	95% CI	*P* value	OR	95% CI	*P* value	OR	95% CI	*P* value
Age category	0.631	0.331–1.206	0.164	0.551	0.254–1.196	0.132	0.782	0.331–1.848	0.575
Gender	1.450	0.601–3.500	0.409	1.181	0.418–3.340	0.754	1.950	0.600–6.331	0.267
Number of siblings	1.218	0.475–3.123	0.681	1.185	0.393–3.570	0.763	1.269	0.363–4.433	0.709
Caregiver level of education	0.872	0.473–1.609	0.661	0.788	0.388–1.599	0.509	1.035	0.440–2.431	0.937
Length of illness	1.141	0.464–2.806	0.773	1.298	0.457–3.688	0.624	0.938	0.273–3.217	0.918
Fever	0.642	0.259–1.592	0.338	1.487	0.458–4.833	0.509	0.219	0.0631–0.759	**0.0166**
Agitation	1.642	0.684–3.940	0.267	0.726	0.248–2.127	0.559	6.222	1.539–25.15	**0.0103**
Altered consciousness	1.659	0.632–4.357	0.304	1.143	0.387–3.376	0.809	3.467	0.695–17.30	0.130
Seizure	9.655	2.049–45.50	**0.00415**	11.59	1.423–94.32	**0.0220**	7.724	0.928–64.26	**0.0586**
Glasgow Coma score^¶^	1	1-1		1	1-1		1	1-1	
Bulging fontanel^¶^	1	1-1		1	1-1		1	1-1	
Cranial nerve palsy^¶^	1	1-1		1	1-1		1	1-1	
Hydrocephalus	1.941	0.307–12.28	0.481	2.200	0.289–16.75	0.447	1.571	0.132–18.66	0.720
Stiff neck	1.056	0.434–2.572	0.904	1.125	0.393–3.219	0.826	0.964	0.293–3.172	0.952
Protein (log base *e*)	0.514	0.245–1.081	**0.08**	0.412	0.127–1.328	0.138	0.564	0.257–1.237	0.153
Glucose	3.437	0.627–18.85	0.155	4.889	0.821–29.10	**0.0812**	1.571	0.132–18.66	0.720
CSF culture	34.29	4.259–276.0	**0.000895**	31.15	3.607–269.1	**0.00177**	39.37	4.250–364.8	**0.00122**

Model 1: normal versus any SNHL; Model 2: normal versus moderate SNHL; Model 3: normal versus severe SNHL. OR: odds ratio. ^¶^Explanatory variable perfectly predicted outcome and estimates could not be obtained by maximum likelihood. The *P* values are based on Wald tests.

**Table 5:** Multivariable ordinary logistic regression.

Variable		Model 1			Model 2			Model 3	
	OR	95% CI	*P* value	OR	95% CI	*P* value	OR	95% CI	*P* value
Age category									
2–11 months	Ref								
12–60 months	0.807	0.239–2.733	0.731	0.324	0.0688–1.523	0.153	10.16	0.696–148.4	0.0901
>61 months	0.107	0.00955–1.201	0.0701	0.242	0.0206–2.850	0.260	8.08*e* − 08		0.995
Gender									
Male	Ref								
Female	2.090	0.652–6.700	0.215	0.853	0.180–4.040	0.841	6.489	0.897–46.96	0.0640
Fever									
No	Ref								
Yes							0.273	0.0321–2.332	0.236
Agitation									
No	Ref								
Yes							12.44	0.851–181.8	0.0654
Seizure									
No	Ref								
Yes	8.289	1.394–49.28	0.0201	11.79	0.994–139.7	**0.0506**	3.363	0.237–47.78	0.370
Glucose									
Normal	Ref								
Elevated				7.973	0.785–80.97	0.0792			
CSF culture							1.487*e* + 09		0.994
No growth	Ref								
Growth	56.83	4.058–795.9	0.00270	62.75	3.623–1,087	**0.00444**			
Protein (log base *e*)	0.499	0.182–1.367	0.176						

Model 1: normal versus any SNHL; Model 2: normal versus moderate SNHL; Model 3: normal versus severe SNHL. OR: odds ratio. The *P* values are based on Wald tests. Only variables significant at *P* value 0.1 level from the univariable models included for each model. Ref is the reference group. Estimates obtained from maximum likelihood.

**Table 6:** Multivariable multinomial and ordered logistic regression.

Variables	Multinomial				Ordered logistic			
	Normal versus moderate		Normal versus severe		Normal versus moderate		Normal versus severe	
	RRR	95% CI	RRR	95% CI	OR	95% CI	OR	95% CI
Age category								
2–11 months	Ref							
12–60 months	4.519	0.622–32.85	0.435	0.108–1.756	0.746	0.223–2.490	0.746	0.223–2.490
>61 months	0.310	0.00790–12.13	0.114*	0.00983–1.326	0.299	0.0437–2.046	0.299	0.0437–2.046
Gender								
Male	Ref							
Female	3.363	0.682–16.59	1.662	0.437–6.318	1.773	0.564–5.571	1.773	0.564–5.571
Fever								
No	Ref							
Yes	0.206*	0.0351–1.209	2.907	0.629–13.44	1.359	0.348–5.311	14.22***	2.304–87.77
CSF culture								
No growth	Ref							
Growth	225.8***	5.987–8.518	51.35***	3.189–826.6	12.45***	2.720–56.99	12.45***	2.720–56.99
Seizure								
No	Ref							
Yes	2.752	0.244–31.03	13.85**	1.353–141.7	24.02**	2.107–274.0	24.02**	2.107–274.0
Agitation								
No	Ref							
Yes	8.165**	1.257–53.02	0.701	0.188–2.612	0.639	0.215–1.897	0.639	0.215–1.897
Coma score								
>8	Ref							
<8					8.108***	1.856–35.41	8.108***	1.856–35.41
Cranial nerve palsy								
No	Ref							
Yes					22.21***	2.652–186.1	22.21***	2.652–186.1

In parentheses, ***P < 0.01, **P < 0.05, and *P < 0.1. RRR: relative risk ratio; OR: odds ratio. The *P* values are based on Wald tests. Estimates obtained by maximum likelihood.
